# Dynamic regulation of TREK1 gating by Polycystin 2 via a Filamin A-mediated cytoskeletal Mechanism

**DOI:** 10.1038/s41598-017-16540-w

**Published:** 2017-12-12

**Authors:** Steven Li Fraine, Amanda Patel, Fabrice Duprat, Reza Sharif-Naeini

**Affiliations:** 1 Department of Physiology and Cell Information Systems, McGill University, 3649 Promenade Sir William Osler, Suite 173, Montreal, H3G0B1 Quebec, Canada; 2 Institut de Pharmacologie Moléculaire et Cellulaire, Université de Nice-Sophia Antipolis, 660 Route des lucioles, Valbonne, 06600 France

## Abstract

Mechanosensing is essential for several physiological functions including touch and pain sensations, osmoregulation, and controlling the myogenic tone of resistance arteries. Understanding how mechanosensitive ion channels (MSCs) are gated can provide important information regarding these processes. We have previously demonstrated that during pathological conditions such as polycystic kidney disease, polycystin 2 (TRPP2) inhibits the activity of potassium-selective MSCs through a filamin A-mediated cytoskeletal effect, and renders tubular epithelial cells susceptible to apoptosis. However, the nature of this cytoskeletal inhibition remains poorly understood. In this study we use a combination of electrophysiology, structured illumination microscopy, and fluorescence recovery after photobleaching (FRAP) to examine the dynamic nature of the TRPP2-mediated cytoskeletal inhibition of the potassium-selective MSC TREK1. Our data indicate that this inhibition of MSC activity occurs through an accelerated cytoskeletal inhibition, and ultimately decreases the open probability of the TREK1 channel. These results shed light on a novel mode of regulation of MSCs gating, which may be at play in several physiological functions.

## Introduction

The ability to perceive mechanical cues from our external or internal environments occurs through a process called mechanotransduction, during which mechanical stimuli are rapidly converted, often in microseconds^1^, into electrical or biochemical signals^2–4^. The rapidity of this process suggests mechanosensitive ion channels (MSCs) are a central part of this effect. These channels are implicated in a series of essential physiological functions such as our senses of touch and hearing^5–8^, as well as the myogenic tone of resistance arteries^9^, baroreflex function^10^, and our ability to regulate hydromineral homeostasis^11–13^. Because of the importance of these functions, the activity of MSCs must be tightly regulated. One of the most important regulators of MSCs is the cytoskeleton^4,14–22^. Both the actin and tubulin cytoskeletons have been shown to modulate mechanosensory processes^4,14–21,23^. In epithelial cells, MSCs have been shown to be under tonic inhibition by both the F-actin and the tubulin cytoskeletons, and disassembly of these networks leads to significant increases in channel activity^9^. In neurons, on the other hand, the cytoskeleton appears to be necessary for the coupling between mechanical stimuli and channel gating. Indeed, rigidification of the cytoskeleton enhances the sensitivity of the mechanotransduction process, whereas cytoskeletal filament depolymerization uncouples mechanical stimuli from channel activation^14,21,23^. These modulatory effects can occur either via a direct interaction between the cytoskeleton and the channel subunits^23,24^, or by the cytoskeleton binding to the membrane near the channel and controlling lateral membrane tension or curvature^25^.

In the case of the F-actin cytoskeleton, its effect on regulating channel activity can differ based on the geometrical arrangement of its filaments. Indeed, it has been proposed that in the presence of the actin crosslinker filamin A (FLNa), fibers are assembled into an orthogonal network^26^ that exerts a greater inhibitory effect on the open probability (Po) of MSCs^9^. This geometry can reduce the membrane radius of curvature and decrease the lateral tension in that domain, therefore effectively reducing channel Po^9^. We have previously reported that when TRPP2 separates from its binding partner polycystin 1, it can recruit FLNa to the plasma membrane, where the latter will indirectly reduce the activity of MSCs present in the membrane^9^. Importantly, this structural rearrangement only reduces the channels’ Po, not their absolute number in the membrane. This was demonstrated using the polymodal MSC TREK1, which can be activated by both mechanical stimuli and intracellular acidosis^27^. When TREK1 is co-expressed with TRPP2, its sensitivity to mechanical stimuli is significantly reduced^18^. However, its membrane expression and response to intracellular acidosis remain intact^18^. The nature of this cytoskeletal inhibition of channel activity, whether it is a static rigidification of the cell cytoskeleton or a dynamic recruitment of cytoskeletal components, remains poorly defined. Understanding the nature of the cytoskeletal modulatory effect is essential for a better understanding of how these channels contribute to health and disease. Here, we use a combination of electrophysiology, imaging with chimeric FLNa, structured illumination microscopy (SIM), and FRAP approaches to examine the nature of the inhibitory effect of FLNa and the F-actin cytoskeleton on TREK1, a potassium-selective MSC.

## Results

### TRPP2 accelerates cytoskeletal inhibition of TREK1

**Figure 1:**
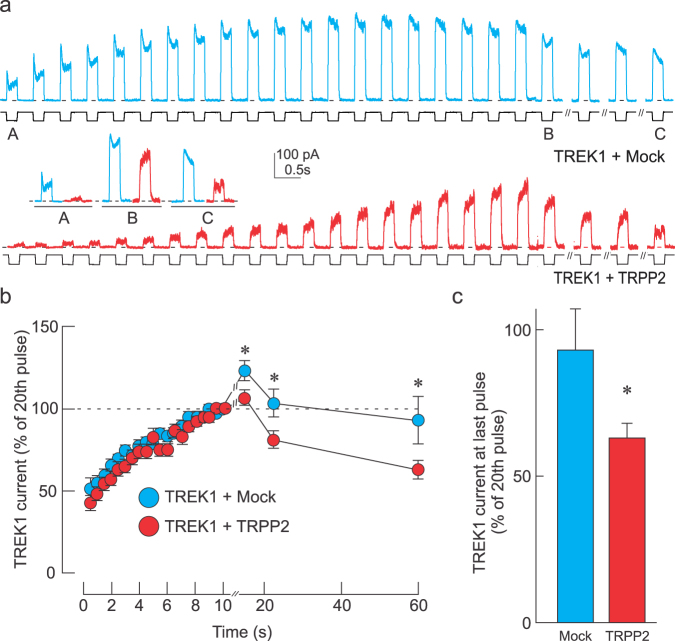
TRPP2 accelerates cytoskeletal inhibition of TREK1. (**a**) Representative traces showing mechanically-elicited TREK1 currents in cells exposed to a −70 mm Hg pressure pulse at 2 Hz and after 2 s, 15 s, and 30 s of rest. Cells were co-transfected with Mock (blue trace) or TRPP2 (red trace)-containing plasmids. Insets labeled A, B and C represent magnifications of the original trace before (A), at the 20^th^ pulse (B), and at the last pulse (C). (**b**) Normalized mechanically-evoked TREK1 current (mean ± s.e.m.) during (0–10 s) and after (10–57 s) the pressure pulse train in TRPP2 (n = 38) expressing and Mock (n = 40) cells. *Indicates significant difference from Mock-transfected group, p < 0.05; Two-way ANOVA with Tukey’s post-hoc test. (**c**) Recovered TREK1 current inhibition at 47 s post pressure pulse train showing a significant increase in TREK1 current inhibition when TRPP2 is expressed. All data expressed as a percentage to the 20th (final) pulse at 10 s. Statistical significance at *p < 0.05, one way ANOVA.

We have previously demonstrated that TRPP2 significantly reduces the mechanical activation of TREK1 through a FLNa-dependent mechanism^18^. Here we examined whether this inhibitory effect occurs through a dynamic process in which the F-actin cytoskeleton (CSK) not only inhibits channel activity, but does so at an accelerated rate. If this is the case, removal of the CSK would enhance channel activity, and if allowed to reform, the F-actin inhibitory effect would set in faster than in the absence of TRPP2. To examine this, we subjected the membrane patch during our cell-attached recordings, to repeated negative pressure pulses (Fig. 1a) to gradually disassemble the underlying F-actin CSK. This approach is based on previous demonstrations of mechanical loads leading to transient cytoskeletal disassembly^20,28,29^. As the number of consecutive pulses increased, we observed an increase in the amplitude of the TREK1 current, which is likely due to the disassembly of the F-actin network. Interestingly, while the amplitude of the TREK1 current elicited by the first pressure pulse was significantly lower in TRPP2- versus Mock-transfected cells, they both reached a plateau of comparable amplitude by the 20th pulse (Fig. 1a), indicating that most of the TRPP2-mediated TREK1 inhibition is relieved by this train of 20 pulses. Furthermore, once the pressure pulses were paused for 2, 15, and 30 seconds, the F-actin cytoskeleton could reform and its inhibitory effect on TREK1 currents reappeared. However, the rate at which this inhibition returned was significantly faster in TRPP2- than Mock-transfected cells (Fig. 1b,c). It is also interesting to note that the first pulse given 2 s after the train produced a current greater than the value at the 20^th^ pulse, suggesting the cytoskeleton kept disassembling during that time (Fig. 1b).

### The accelerated TREK1 inhibition requires filamin A

**Figure 2:**
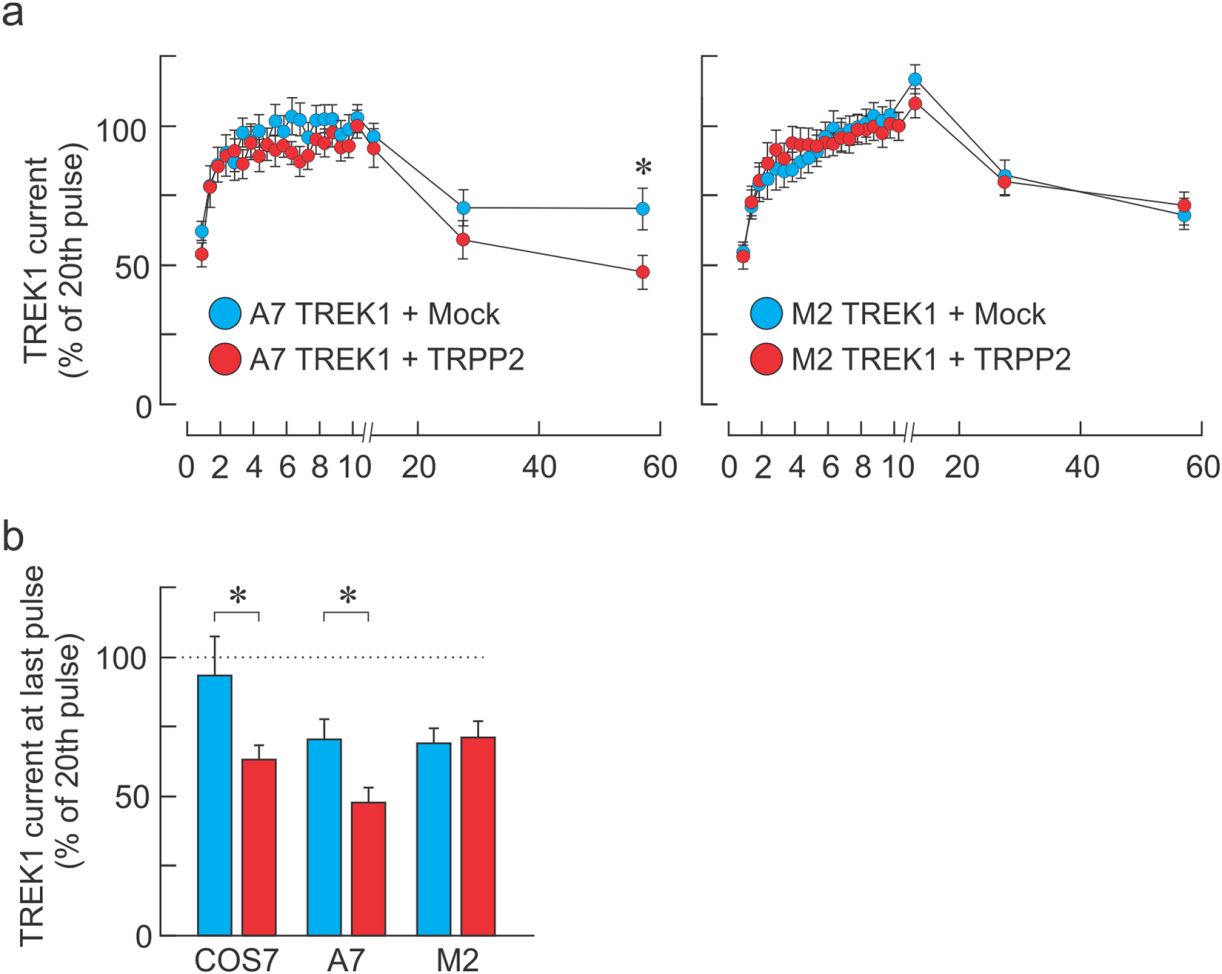
TRPP2-elicited acceleration of cytoskeletal inhibition requires Filamin A. (**a**) Normalized mechanically-evoked TREK1 current (mean ± s.e.m.) during (0–10 s) and after (10–57 s) a train of negative pressure pulses (−70 mm Hg at 2 Hz) in A7 (left panel) or FLNa knockout cells (M2, right panel) transfected with a Mock (blue, n = 23 and 51 in A7 and M2 cells, respectively) or TRPP2 (red, n = 17 and 38 for A7 and M2 cells, respectively) plasmid. *Indicates significant difference from Mock-transfected group, p < 0.05. Two-way ANOVA with Tukey’s post-hoc test. (**b**) Recovered TREK1 current inhibition 47 s after ending the pulse train in COS7, A7 and M2 cells. All data expressed as a percentage to the 20th (final) pulse at 10 s. Statistical significance at *p < 0.05, One Way ANOVA.

We have previously shown that most, if not all, of the inhibitory effect of TRPP2 on the activity of MSCs is via FLNa^9,18^. We examined whether the accelerated rate of inhibition of TREK1 currents once the F-actin cytoskeleton is allowed to reform depended on the presence of FLNa. When FLNa-expressing A7 cells were transfected with TREK1 and Mock or TRPP2, we observed a similar effect of TRPP2: briefly, repeated pulses of negative pressure led to a removal of the cytoskeletal inhibitory effect, which promptly resumed once the stimuli stopped (Fig. 2a). However, the rate at which the inhibition resumed was significantly faster in A7 cells transfected with TRPP2 than Mock. Interestingly, when the same procedure was applied to FLNa-deficient cells (M2 cells), the recovery of the cytoskeletal inhibitory effect was the same in the two conditions (Fig. 2a,b). This indicates that the accelerated rate at which inhibition occurs was dependent on the presence of FLNa.

### TRPP2 recruits filamin A to the plasma membrane

**Figure 3:**
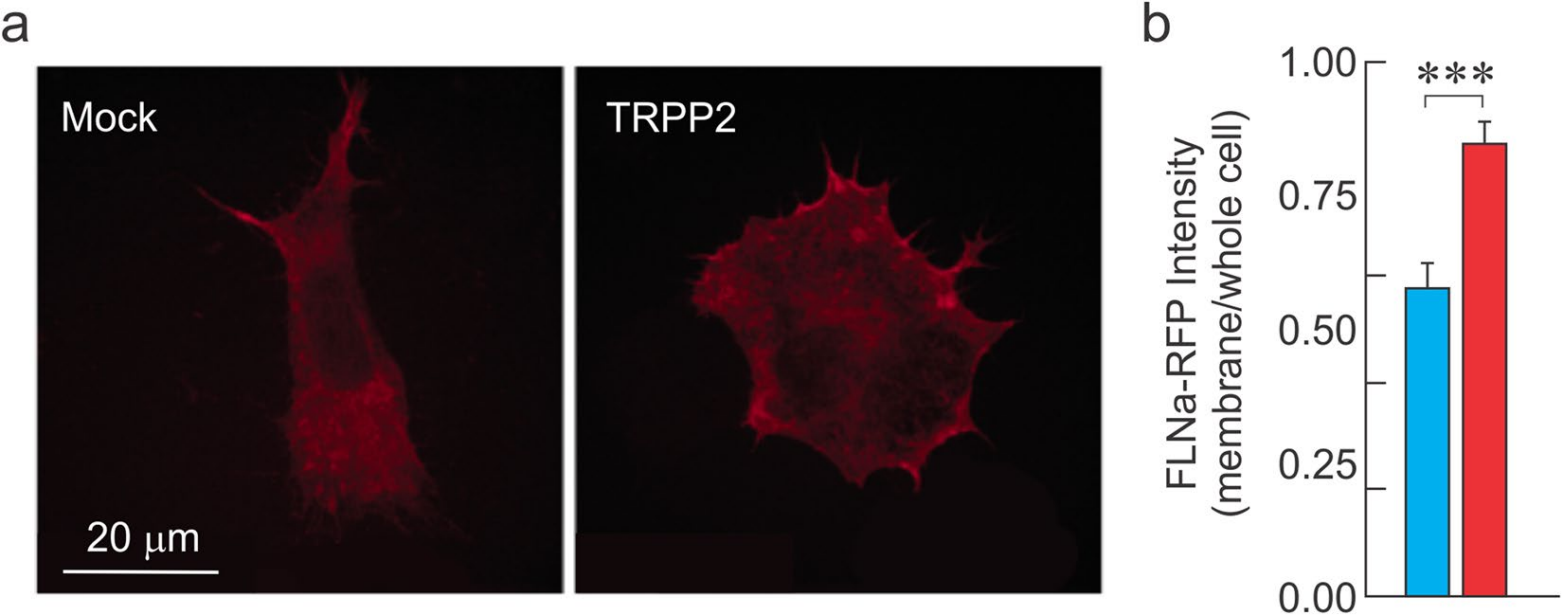
TRPP2 increases the membrane-targeting of Filamin A. (**a**) M2 cells stably expressing chimeric fluorescent FLNa-RFP (termed S8 cells) allow the subcellular tracking of FLNa. Examination of FLNa membrane expression using S8 cells transfected with Mock (left) or TRPP2 (right panel)-expressing plasmid. (**b**) FLNa-RFP fluorescence intensity expressed as membrane signal normalized to whole cell signal in S8 cells transfected with Mock (blue) or TRPP2 (red) plasmids. n = 10 cells per condition. Statistical significance at ***p < 0.001 Unpaired *t* test.

We have previously speculated that the mechanism of TRPP2-induced inhibition of MSC activity occurs through a reorganization of the F-actin CSK via the recruitment of FLNa to the cell membrane, leading to a decrease in channel open probability^9,18^. For FLNa to produce this CSK rigidification and the reduced MSC activity observed through the cell-attached recordings, this actin-binding protein must exert its cross-linking effects near the plasma membrane, in a TRPP2-dependent manner. Although previously speculated, this TRPP2-mediated membrane targeting of FLNa was never demonstrated. To monitor the subcellular localization of FLNa, we used a chimeric form of the protein in which it is fused to a red fluorescent protein (FLNa-RFP; gift from the Stossel lab). To examine the localization of FLNa independently of the endogenous protein, we generated a stable cell line by expressing FLNa-RFP in the M2 cell background (termed S8 cells, Fig. 3a). We examined the membrane localization of FLNa-RFP in S8 cells transfected with TRPP2 or a Mock plasmid. Our data demonstrate that in S8 cells transfected with TRPP2, the proportion of FLNa-RFP observed at the membrane is significantly larger than in Mock-transfected cells (Fig. 3a,b).

### TRPP2 recruits FLNa to reorganize the F-actin cytoskeleton

**Figure 4:**
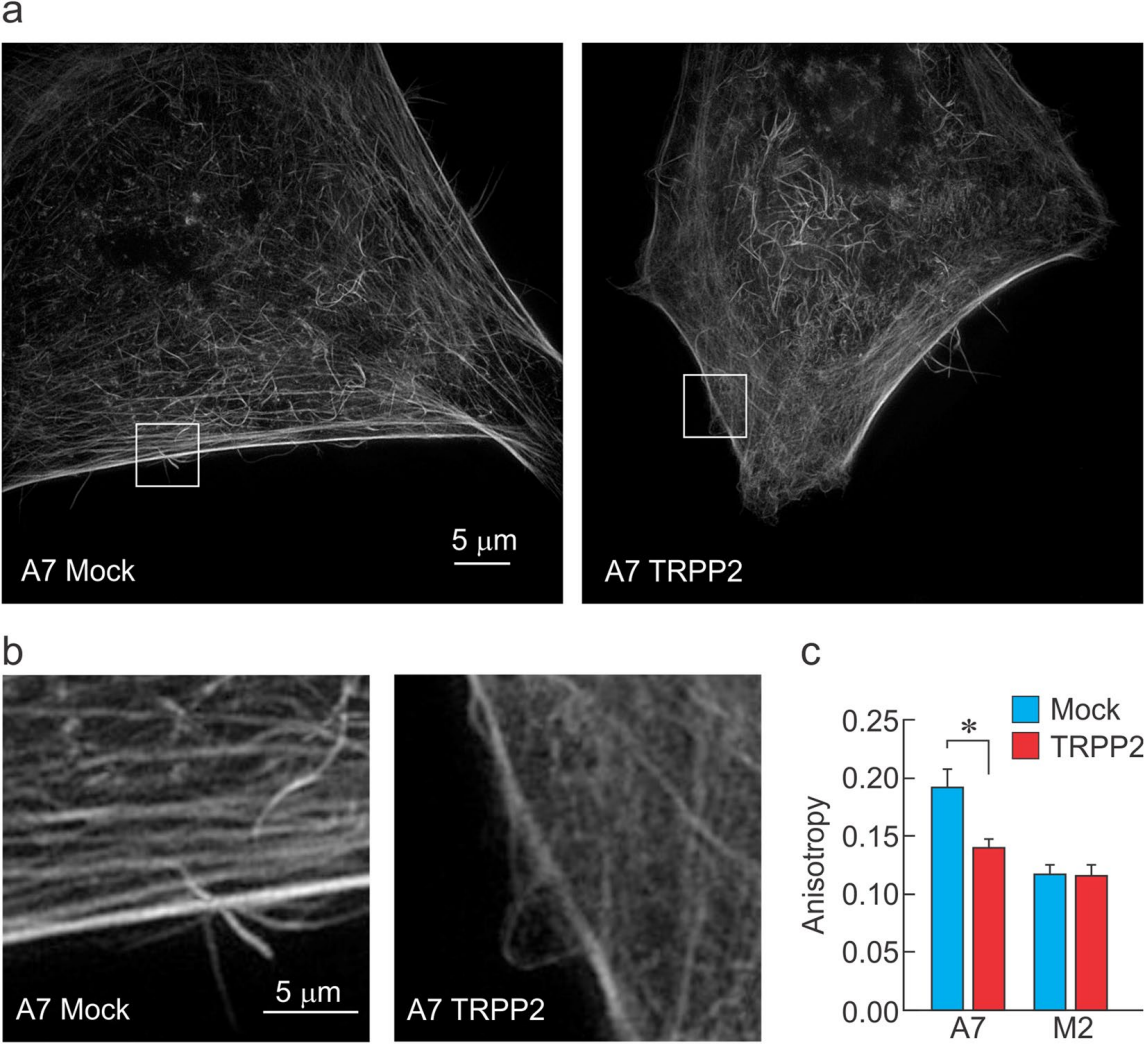
TRPP2 requires filamin A to reduce the anisotropy of F-actin filaments. (**a**) Representative F-actin staining images obtained via ultra-structured illumination microscopy in A7 cells transfected with Mock (left) or TRPP2 (right) plasmids. (**b**) Magnification of images in (**a**) indicates a loss of interdependency of fiber orientation at the level of the subcortical cytoskeleton. (**c**) Mean (±s.e.m.) anisotropy level in A7 and M2 cells transfected with Mock or TRPP2. *Indicate significant different with p < 0.05; unpaired *t*-test. n = 15, 15, 15, and 13 for A7-Mock, A7-TRPP2, M2-Mock and M2-TRPP2, respectively.

Filamin A is an F-actin crosslinker that reorganizes the cytoskeleton into an orthogonal structure^26^. We hypothesized that its recruitment to the cell cortex would impact the organization geometry of the F-actin filaments, therefore decreasing the anisotropy of F-actin fiber arrangement. To examine the organization of the actin meshwork at the level of the actin cytoskeleton, we used structured illumination microscopy to image A7 and M2 cells expressing TRPP2 or a Mock plasmid. In the presence of TRPP2, we observed a decrease in anisotropy in the F-actin subcortical cytoskeleton (Fig. 4a–c). This indicates that the F-actin filaments are less directionally dependent on each other, thus suggesting reorganization from a more parallel to a more orthogonal arrangement. Furthermore, this phenomenon only occurs in A7 cells and not M2 cells, confirming that TRPP2 depends on FLNa to reorganize the F-actin cytoskeleton.

### TRPP2-mediated acceleration of cytoskeletal inhibition is not mediated by a faster recruitment of F-actin filaments

**Figure 5:**
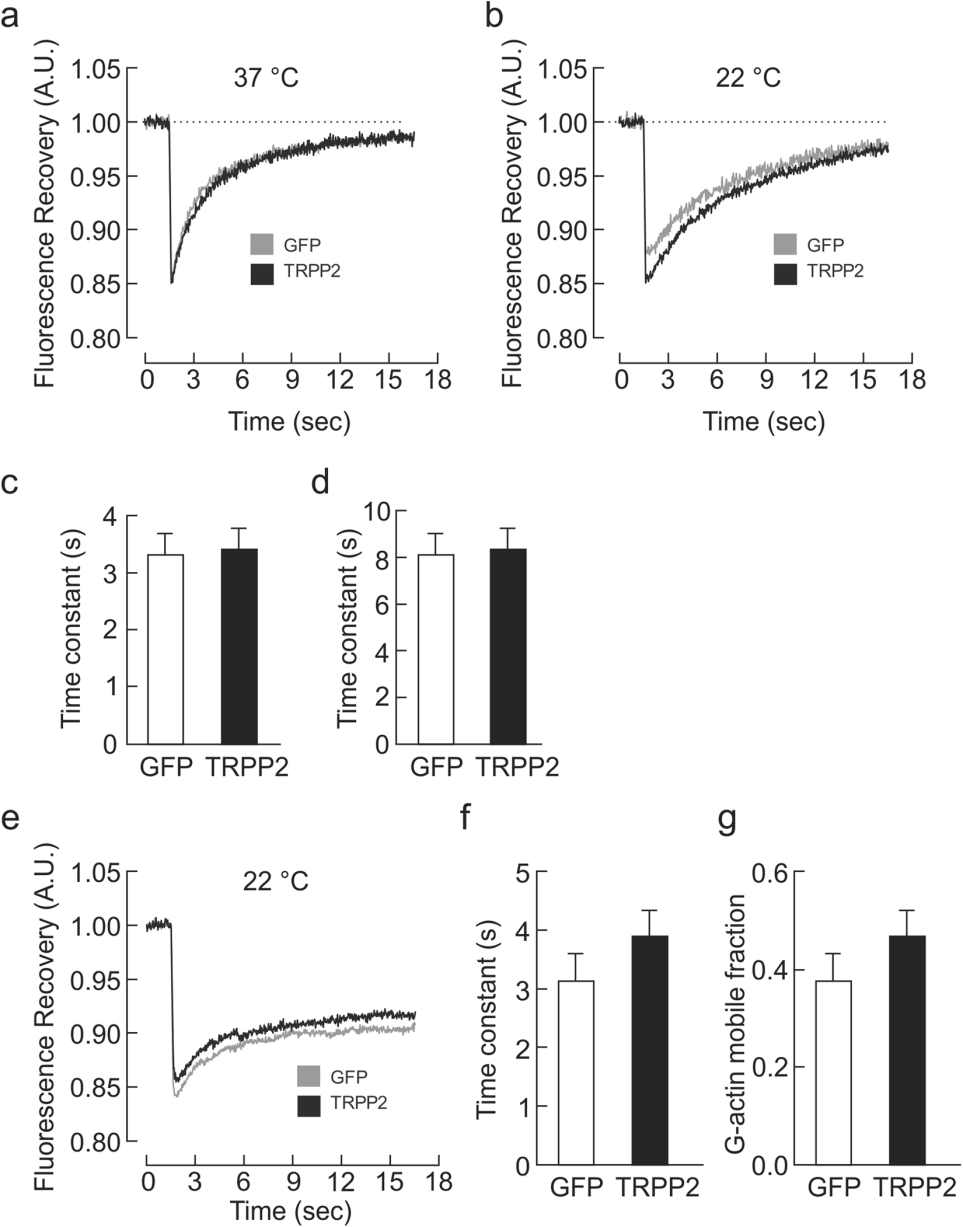
TRPP2-mediated acceleration of cytoskeletal inhibition is not mediated by a faster recruitment of F-actin filaments. Averaged traces showing the recovery of LifeAct-RFP fluorescence signal following photobleaching at t = 1.5 s in COS7 cells expressing TRPP2 (n = 34) or Mock (n = 24) plasmids at 37 °C (**a**) and 22 °C (**b**). Respective mean ( ± s.e.m.) recovery time constants at 37 °C (**c**) and at 22 °C (**d**). (**e**) Averaged traces showing the recovery of GFP-Actin signal following photobleaching at t = 1.5 s in COS7 expressing TRPP2 (n = 21) or Mock (n = 21) plasmids at 22 °C with respective (**f**) mean ( ± s.e.m.) recovery time constant and (**g**) mean (±s.e.m.) mobile fraction. No significant differences were observed using an unpaired *t* test.

The accelerated TREK1 current inhibition in the presence of TRPP2 led us to hypothesize that F-actin filaments are recruited to the actin cytoskeleton at a faster rate in the presence of TRPP2 than in its absence. To examine the actin dynamics at the cytoskeletal membrane, we performed FRAP experiments in COS7 cells co-transfected with LifeAct-RFP^30^ to visualize F-actin filaments, and either TRPP2 or Mock plasmids. At physiological temperatures (37 °C), we saw no difference in the recruitment of F-actin filaments to the subcortical cytoskeleton (Fig. 5a,c). To tease out any small time differences in FRAP that may not be observable at 37 °C, we slowed the kinetics by lowering the temperature to 22 °C and showed that no observable differences in FRAP were found in COS7 cells expressing TRPP2 vs mock plasmids (Fig. 5b,d). To examine whether the trafficking of individual G-actin monomers is affected by TRPP2 expression, we co-transfected COS7 with GFP-actin as well as TRPP2 or Mock plasmids and examined the FRAP kinetics at 22 °C (Fig. 5e,f). Our data indicate that the trafficking of G-actin monomers is unchanged by TRPP2 expression (Fig. 5f). We next considered whether TRPP2 mediates cytoskeletal inhibition of TREK1 currents by restraining actin monomers at the level of the cell membrane, thus limiting the fraction of actin monomers that are actively turned over (i.e. the mobile fraction). If this was the case, then less actin would need to be recruited to the membrane for effective cytoskeletal inhibition. Therefore, the rate of TREK1 current inhibition would be faster irrespective of the rate of actin recruitment. However, examination of the magnitude of the mobile fraction extracted from FRAP curves of GFP-actin-transfected COS7 cells indicated no significant difference between TRPP2- or Mock-transfected cells (Fig. 5g).

## Discussion

Mechanosensitive ion channels are a central part of several physiological processes, such as our senses of touch and pain^5,6,31^. In pain-sensing neurons (nociceptors), painful mechanical stimuli are converted into depolarizing membrane potentials by non-selective cationic MSCs, which produce a depolarization large enough to activate voltage-gated ion channels at the action potential trigger zone^31^. This depolarizing effect can be blunted by the simultaneous activation of potassium-selective MSCs that tend to hyperpolarize the nerve terminal in response to mechanical stimuli. Thus mice lacking the TREK1 channel show a lower threshold to mechanical stimuli^32^. The contribution of these channels is also highlighted during inflammatory pain, in which inflammation causes a decrease in the expression of TREK1 and is associated with reduced thresholds to mechanical stimuli^33,34^. Alternatively, the expression level of these potassium-selective MSCs may be unaltered, but their gating by mechanical stimuli can be impaired. This has been demonstrated for epithelial cells of the proximal convoluted tubules which, during the cystogenesis that occurs as a result of polycystic disease, get compressed and undergo apoptosis. These cells normally express a functional mechanosensitive TREK2 ion channel which, during autosomal dominant polycystic kidney disease (ADPKD), becomes inhibited through a TRPP2-FLNa-dependent mechanism. It is therefore essential to better understand how the gating of MSCs can be modulated by accessory proteins such cytoskeletal elements.

In this study, we examined the mechanisms through which TRPP2 can block the mechanical activation of TREK1. More specifically, we examined the dynamics of this inhibition and the contribution of the F-actin CSK to this inhibition. To examine the dynamic nature of the CSK inhibition, we designed an experimental approach derived from previously-published observations^20^. We reasoned that if the cytoskeletal inhibition of TREK1 were a static phenomenon, then removing it by continuously stimulating the membrane with mechanical stimuli would prove to be difficult, and once the cytoskeleton was damaged, it would take a long time for it to reassemble and re-impose its inhibitory effect. However, if the inhibition were dynamic, we should be able to easily remove it and observe the time course at which it comes back. Our data demonstrates that within 10 seconds of stimulation at 2 Hz, we can remove most, if not all, of the inhibition caused by TRPP2 expression. Interestingly, the rate at which this inhibition returns is significantly faster in cells expressing TRPP2. This accelerated return of inhibition depends on the expression of FLNa in the cell, and is likely due to the membrane-targeting effect of TRPP2 on FLNa. Indeed, the subcellular localization of FLNa indicates a significant shift to the plasma membrane when TRPP2 is expressed. Filamin A changes the geometry of the F-actin network to a more orthogonal structure, thereby decreasing the directional dependence of fibers. This anisotropy can be measured by using super-resolution imaging of phalloidin-stained actin filaments. Our data indicate that A7 cells transfected with TRPP2 have a decrease in anisotropy compared to Mock-transfected cells. This decreased anisotropy is absent in TRPP2-transfected M2 cells, compared to Mock-transfected M2 cells.

We examined whether the accelerated recovery of inhibition was due to an increase in speed at which F-actin filaments are turned over. To test this hypothesis, we performed FRAP experiments on plasma membranes of Mock and TRPP2-transfected cells, and observed no difference in the two groups. The absence of a difference in FRAP time course between the two groups can be explained by several factors: (1) The cytoskeletal inhibition could be occurring within the microdomains where TREK1 is located instead of being present throughout the membrane. (2) Alternatively, the absence of a FRAP could be due to the large size of the field of view. Indeed, to efficiently photobleach a circular field, the minimum zone diameter had to be 6.53 μm^2^ with the circle’s widest region on the membrane. It is therefore possible that the FRAP effect occurred only in a restricted area under the membrane and photobleaching a larger area diluted fluorescence recovery. Finally, (3) it is possible that the absence of a faster FRAP is accurate, and the F-actin filaments in Mock or TRPP2-transfected cells reassemble at the same rate, only that in TRPP2-expressing cells, this reassembly occurs with an orthogonal geometry, which has a more efficient inhibitory effect on MSC gating.

Because TREK1 is widely expressed in the nervous system, and has several mechanosensory functions, understanding how its gating is regulated may prove useful in the development of future therapeutic tools aimed at reducing its inhibition by the cytoskeleton.

## Methods

### Cell Culture

The fibroblast-like COS-7 cell line derived from African green monkey kidneys were used in addition to the human melanoma derived M2 and A7 cell lines. Cos-7 cells were maintained with Dulbeco’s Modified Eagle’s Growth Medium (DMEM; Wisent) supplemented with 10% Fetal Bovine Serum and 1% penicillin/streptomycin. M2 and A7 cells were maintained in Eagle’s Minimum Essential Medium (EMEM; Wisent) supplemented with 2% Newborn Calf Serum, 8% Fetal Bovine Serum, and 1% penicillin/streptomycin. A7 cells were additionally grown with 200 μg/mL of geneticin (Wisent) to maintain stable expression of Filamin A. One day prior to transfection, cells were plated at ~50% confluency onto 35 mm plastic dishes. All constructs were transfected using FuGene 6 (Promega) according to manufacturer’s instructions using 2 μg of DNA. Cells co-transfected with two plasmids received 1 μg of each plasmid. One day following transfection, cells were plated onto 35 mm glass bottom dishes. All recordings were performed 48 hours after transfection.

### Electrophysiology

Cell-attached recordings were performed on transiently transfected COS7, M2, and A7 cells. The extracellular recording medium contained 155 mM KCl, 5 mM, EGTA, 3 mM MgCl2, and 10 mM HEPES (pH 7.22, 310 mOsm). The pipette solution contained 150 mM NaCl, 5 mM KCl, 1 mM CaCl2, and 10 mM HEPES (pH 7.4, 310 mOsm). The pipette solution also contained 10 mM Tetraethyl-ammonium (TEA), 5 mM 4-Aminopyridine (4AP), and 10 μM glibenclamide to block contaminating potassium channels. After achieving a gigaseal in the cell-attached configuration and voltage clamping at 0 mV, membrane patches were stimulated with repeated 200 ms pressures pulses (−70 mmHg at 2 Hz) through the recording electrode/pipette setup using a high-speed pressure clamp device (ALA Scientific Instruments). After the 10 s stimulation, the membrane was allowed to rest for 2 s, 15 s, and then 30 s before subsequent pressure pulses were given. All recordings were performed with an Axon MultiClamp 700B amplifier (Molecular Devices) using non-coated fire polished glass pipettes (1.4–2.4 MΩ). Clampex 10.3 (Molecular Devices) was used for data acquisition. Data analysis and figure preparation was performed using Clampfit 10.3 (Molecular Devices), Microsoft Excel, and Prism 6 (GraphPad Software).

### Fluorescence Recovery After Photobleaching (FRAP)

FRAP experiments were performed on a spinning disk confocal microscope (Quorum WaveFX, Leica) using a 63x objective. A 100 ms laser pulse was used to photobleach a 6.53 μm^2^ area on the cell membrane. Images were captured with MetaMorph (Molecular Devices) before and for ~17 s after photobleaching to acquire fluorescence recovery at the cell membrane. Data was corrected for the overall bleaching of the background signal resulting from image acquisition. Fluorescence data was fit to an exponential function to extrapolate the recovery time constant using Prism 6 (GraphPad Software). The mobile fraction (*MF*) was calculated by taking the ratio of fluorescence signal that recovered post photobleaching (*F*_*final*_ − *F*_*post*_) to the the total fluorescence signal that was photobleached (*F*_*pre*_ − *F*_*post*_).

$$MF=\frac{{F}_{final}-{F}_{post}}{{F}_{pre}-{F}_{post}}$$

where *F*_*pre*_ is the fluorescent signal pre-photobleaching; *F*_*post*_ is the fluorescent signal immediately post-photobleaching; and *F*_*final*_ is the final fluorescent signal.

### Filamin A Subcellular Localization

The S8 cell line was generated by stably transfecting M2 cells with a red fluorescent form of Filamin A (Filamin A-RFP; received from the Stossel Lab). S8 cells transiently transfected with TRPP2 or a mock control were fixed with 4% paraformaldehyde and then treated with WGA-350 to label the plasma membrane. Images were captured at 63x on a spinning disk confocal microscope (Quorum WaveFX, Leica). The WGA-350 signal was used as a mask to determine the Filamin A-RFP signal at the membrane, which was then normalized to the overall Filamin A-RFP cell intensity. ImageJ (National Institutes of Health) was used to perform data analysis.

### Structured Illumination Microscopy (SIM)

Transiently transfected M2 and A7 cells were fixed with 4% paraformaldehyde and treated with phalloidin-488 (Cytoskeleton) to stain actin filaments. Super resolution images were collected using an OMX V4 microscope (Applied Precision/GE Deltavision) using a 100x objective. OMX and softWoRx softwares (Applied Precision/GE Deltavision) were used for image acquisition and structured illumination reconstruction, respectively. Analysis of SIM data was performed using ImageJ (NIH) and the plugin FibrilTool to calculate anisotropy, the property of being directionally dependent, amongst actin filaments^35^. The cell anisotropy values were measured by analyzing 4μm thickness of actin staining just under the plasma membrane.

### Statistical analysis

Results are represented as mean ± SEM. Statistical significance was tested using unpaired t-tests for comparison of means, or two-way ANOVA for comparisons of groups over time. Differences were considered significant for p < 0.05 (*).
